# Intensity Modulated High Dose Rate (HDR) Brachytherapy Using Patient Specific 3D Metal Printed Applicators: Proof of Concept

**DOI:** 10.3389/fonc.2022.829529

**Published:** 2022-02-10

**Authors:** James J. Sohn, Mitchell Polizzi, Sang-Won Kang, Woo-Hyeong Ko, Yong-Hyun Cho, Keun-Yong Eom, Jin-Beom Chung

**Affiliations:** ^1^ Department of Radiation Oncology, Virginia Commonwealth University, Richmond, VA, United States; ^2^ Department of Radiation Oncology, Seoul National University Bundang Hospital, Seongnam, South Korea; ^3^ Department of Anesthesiology and Pain Medicine, Seoul Sungsim General Hospital, Seoul, South Korea

**Keywords:** HDR brachytherapy, IMBT, 3D metal printing, patient-specific, tandem applicator

## Abstract

**Purpose:**

In high-dose-rate (HDR) brachytherapy, an anisotropic dose distribution may be desirable for achieving a higher therapeutic index, particularly when the anatomy imposes challenges. Several methods to deliver intensity-modulated brachytherapy (IMBT) have been proposed in the literature, however practical implementation is lacking due to issues of increased delivery times and complicated delivery mechanisms. This study presents the novel approach of designing a patient-specific inner shape of an applicator with 3D metal printing for IMBT using an inverse plan optimization model.

**Methods:**

The 3D printed patient-specific HDR applicator has an external shape that resembles the conventional brachytherapy applicator. However, at each dwell position of the HDR source, the shielding walls in the interior are divided into six equiangular sections with varying thicknesses. We developed a mathematical model to simultaneously optimize the shielding thicknesses and dwell times according to the patient’s anatomical information to achieve the best possible target coverage. The model, which is a bi-convex optimization problem, is solved using alternating minimization. Finally, the applicator design parameters were input into 3D modeling software and saved in a 3D printable file. The applicator has been tested with both a digital phantom and a simulated clinical cervical cancer patient.

**Results:**

The proposed approach showed substantial improvements in the target coverage over the conventional method. For the phantom case, 99.18% of the target was covered by the prescribed dose using the proposed method, compared to only 58.32% coverage achieved by the conventional method. For the clinical case, the proposed method increased the coverage of the target from 56.21% to 99.92%. In each case, both methods satisfied the treatment constraints for neighboring OARs.

**Conclusion:**

The study simulates the concept of the IMBT with inverse planning using the 3D printed applicator design. The non-isotropic dose map can be produced with optimized shielding patterns and tailored to individual patient’s anatomy, to plan a more conformal plan.

## Introduction

High dose rate (HDR) brachytherapy is a treatment option for cervical cancer patients, which is either paired with external beam radiation therapy (EBRT) or delivered alone (1–3). Compared to the advances in EBRT, such as image-guided radiation therapy (IGRT) and volumetric-modulated arc therapy (VMAT), analogous innovations in brachytherapy are lacking. The limiting factor for brachytherapy, as in most radiotherapy, is the dose delivered to surrounding organs at risk (OARs), however brachytherapy has been limited due to the reduced degrees of freedom for dose modulation, with the main adjustable parameters being dwell time and position (4). Since conventional brachytherapy applicators typically include a single, central lumen where the radioactive source resides, the dose profiles tend to be highly isotropic (5). The distribution of dose surrounding single lumen applicators limit the ability to satisfy OAR dose constraints while simultaneously delivering the prescribed dose to the tumor (6). The drawbacks of these applicators are especially apparent in cases where the tumor is large, laterally extended, and/or anisotropically distributed (7).

In general, the existing approaches for treating the irregular-shaped tumor and/or adjacent to the OAR aim to create a more conformal dose profile by supplementing the intracavitary tandem with interstitial brachytherapy. Such approaches include using a modified tandem and ring applicators in which the ring also acts as a template for interstitial needles (8). These approaches are common in the clinic but still suffer the drawbacks of being able to create dose distribution through only modulating dwell time and dwell positions. The placing of interstitial needles is also very clinician-dependent and requires training and practice to retain sufficient clinical ability.

Based on the review paper recently published in 2019, there are have been many attempts with different techniques and prototypes of the applicators for treating irregular shaped tumor and/or bulky disease that is adjacent to OAR (9). The authors categorized the various methods of intensity-modulated brachytherapy (IMBT) into two broad branches, static-shielding and dynamic-shielding, each with a sub-category for either a shielded source or shielded applicator. In brief, IMBT is achieved through one of these four ways. In static shielded source IMBT, the source is shielded and the source remains still (e.g., CivaSheet) (10). The static shielded applicator is one of the first examples of IMBT and is most commonly thought of with the shielded ovoid or shielded cylinder. The dynamic shielded source, while not common, includes sources made of Yttrium 90, but only half of the angular section, with the other half being constructed on tungsten. The source itself can be rotated to modulate the dose. For the dynamic shielded applicator, the source is unshielded, but a shielded applicator is rotated around the source. One of the modalities using dynamic shielded applicator, is the paddle-based rotating-shield (P-RSBT) brachytherapy that uses an electronic brachytherapy source. This methodology also has proposed using MLC-like tungsten paddles that shield the radiation field along various angles. As with many of the dynamic IMBT modalities, this suffers from complex mechanical and electrical mechanisms, however the studies showing the theoretical benefit are promising.

Despite the improvements offered by these methods, many of these methods are complicated and may further increase the invasiveness or time of delivery for brachytherapy. For instance, the rotating shield brachytherapy requires a complicated mechanical system that is difficult to perform quality assurance for the patient treatment plans. It requires longer treatment time and therefore is not practical to be used in the current clinical setting. Moreover, these approaches are still limited by the isotropic dose distribution of the radioactive source (11). None of the approaches provide an applicator specific to a patient’s individual anatomy (e.g., the tumor size and position in relation to the position of the surrounding OARs), which means that the full capacity of delivering continuous and most conformal radiation plan possible has not yet been reached. Therefore, a novel transformative approach is needed to fully unleash the potential of HDR brachytherapy.

In this paper, we present our novel concept of utilizing a 3D printed high-Z metal tandem applicator to achieve a patient-specific intensity-modulated HDR brachytherapy, which could improve target coverage compared to existing brachytherapy treatments while further sparing the OARs. The designed applicator would have a unique cross-section with different shielding thicknesses of the internal shape per angle as well as each dwell position based on a patient’s anatomical information to produce a non-isotropic radiation field. In order to validate the proposed method, we developed the inverse-plan-based optimization model to generate the model parameters by calculating the dose and transmission rate at each dwell time and position. To demonstrate the theoretic benefit of the 3D printed high-Z tandem, we produced 2D simulations to compare the 3D metal printed tandem’s optimized dose distribution compared to an unshielded and un-optimized single channel implant.

## Method and Materials

### Method Overview

**Figure 1:**
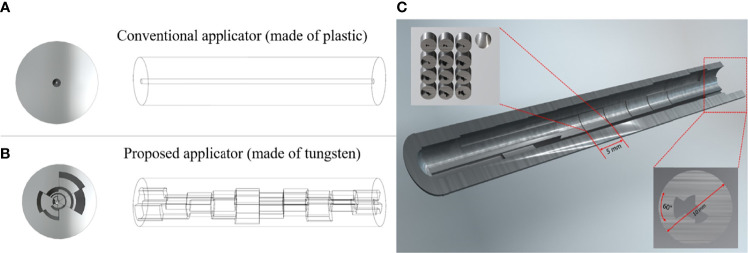
A conceptual diagram of our prosed applicator that differs with the conventional applicator. **(A)** is the conventional HDR method, and **(B)** is the proposed HDR method, and **(C)** is the inside view of the whole applicator. Each dwell position has 5 mm length and the cross-section has a different shielding thickness per 60-degree angle.

The main feature of the proposed approach is a circumferentially different cross-section of shielding segments per each 60-degree angle and each dwell position distributed along a central longitudinal axis. The intrauterine tandem diameter was fixed at 1.2 cm, to allow ample shielding material, while also minimizing the size of the tandem for ease of insertion into the cervix. Due to the size of the tandem, it will necessitate the use of appropriate anesthesia for the duration of the procedure. For the shielding, tungsten was chosen to sufficiently attenuate the photon energies from an Ir-192 source while maintaining compatibility with magnetic resonance (MR) imaging (12). **Figure 1** depicts a conceptual diagram of the difference between a conventional and our proposed applicator. In order to determine the optimal thickness of the shielding as per target and OARs, we developed a mathematical model based on TG-43 to calculate the transmission rates of the applicator and dwell time of the source (13, 14). Individual components of the optimization are outlined in further detail in the following sections.

### Dose Calculation Model

In order to calculate the radiation dose rate, a 2D isotropic point source dose rate calculation formulation suggested by TG-43 was utilized. Specifically, the dose rate $\dot{D}(\vec{r})$ at a voxel at a position $\vec{r}$ from a point source at position ${\vec{r}}_{0}$ is calculated as,


(1) $$\dot{D}(\vec{r})=S_{K}\cdot \Lambda \cdot \left(\frac{\left||{\vec{r}}_{0}|\right|}{\left||\vec{r}|\right|}\right)2\cdot g_{P}(\vec{r})\cdot \phi_{an}(\vec{r})$$

where *S_K_* is the air-kerma strength (units µGym^2^h^-1^), Λ is the dose rate constant in water, $g_{P}(\vec{r})$ is the radial dose function of the point source, and $\phi_{an}(\vec{r})$ is the 1D anisotropy function. The 1D anisotropy function was chosen due to the fact that at clinical distances with the additional shielding from the applicator, the angular dependence of the 2D anisotropy function is minimal, with changes in the anisotropy being largely attributed to the variation in the tungsten material present. The volume of interest is then discretized into voxels with resolution [*r_x_* mm × *r_y_* mm × *r_z_* mm] and index *i*. The dose rate in voxel *i* is subsequently induced by the *j^th^* source, denoted by ${\dot{D}}_{l}^{J}$ . Assuming there are *N_s_* source positions located along the cylinder separated by an equal distance ds and the cylinder consists of *N_t_* pieces of shielding, the total dose received by the *i^th^* voxel is given by


(2) $$D_{i}=\sum_{j=1}^{N_{S}}{\dot{D}}_{l}^{J}\cdot t_{j}\cdot T_{g(i,j)}^{\alpha (i,j)}$$

**Figure 2:**
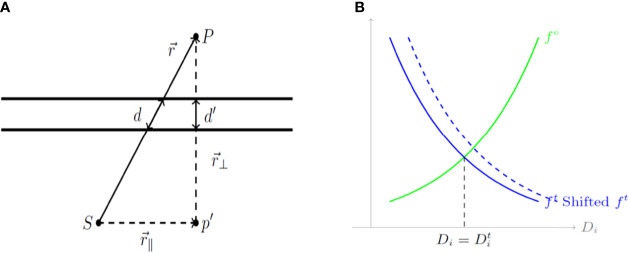
**(A)** A geometrical diagram of a dose calculation. **(B)** Cost functions for organs at risk (OARs) and the tumor based on the calculated dose Di. d, a length of shield; S, source; P, the point of interest; (r_∥ )^⇀^, parallel component; and (r_⊥ )^⇀^, perpendicular component.

Where $T_{g(i,j)}^{\alpha (i,j)}$ is the transmission rate of a given piece of shield indexed by *g*(*i* ,*j*) ranging from 1 to *N_t_*, which is a function that determines which shield a ray originating at the *j^th^* source will cross in traveling to the *i^th^* voxel. *α*(*i*, *j*) is a constant representing the ratio $\frac{\left||\vec{r}|\right|}{\left||{\vec{r}}_{⊥}|\right|}$ , which reflects the effect of the thickness of the shield on the ray passing through it, as shown in **Figure 2A**. The transmission factor of the shield is inversely proportional to its thickness, a factor that guides the design of the cylinder before the 3D printing process. **Figure 2A** is a geometrical representation of dose calculation where the vector $\vec{r}$ that travels through a length of shield *d* from source *S* to the point of interest *P* is composed of parallel and perpendicular components, ${\vec{r}}_{}$ and ${\vec{r}}_{⊥}$ respectively. To determine the dwell time for each dwell position and the transmission rate for each shielding portion, the following optimization model is utilized. In the optimization model, *N_p_* dwell position is assumed and t ∈ 𝒳 represents the vector of dwell times, and T ∈ 𝒴 represents the vector of transmission rates, where


$$X=\{t\in R^{N_{P}}|t_{i}\in [0,+\infty ],i=1,2,\ldots ,N_{p}\}\text{ and }Y=\{T\in R^{N_{s}}|T_{i}\in [l,u],i=1,2\ldots ,N_{s}\}$$

In the model, *l*, *u* ∈ (0,1) are the lower and upper bounds of the transmission rate, which correspond to the thickness of each shielding sector. Considering the design limitations of the shield, *l* = 0.38 and *u* = 0.90 were set corresponding to thicknesses of 0.5 mm and 4.61 mm, respectively. Subsequently, 0 = {1,2,…,*N*_0_} represents the set of indexed OARs to be considered in the optimization method. 𝒱_o_ ⊂ 𝒱 represents the set of voxels making up the *o*-th OAR, while 𝒱*_t_* ⊂ 𝒱 represents the set of voxels in the high-risk clinical target volume (HR_CTV). The cost function for this optimization model is defined as,


(3) $$F(t,T)=\sum_{o\in 0}F_{o}(t,T)+F_{c}(t,T)\sum_{o\in 0}\sum_{i\in V_{o}}f_{i}^{o}(t,T)+\sum_{i\in V_{i}}f_{i}^{t}(t,T)$$

The first term in Equation 3 corresponds to the costs for OARs with the given configuration of dwell times *t* and the transmission rates T while the second term corresponds to the cost for the tumor. Additionally, the cost function at the voxel level for the OARs is defined according to Equation 4 as below,


(4) $$f_{i}^{o}(t,T)=\exp\left(\frac{D_{i}(t,T)-D_{i}^{t}}{C}+S_{o}\right)$$

The cost function at the voxel level for the tumor is defined according to Equation 5 as below,


(5) $$f_{i}^{t}(t,T)=\exp\left(\frac{D_{i}^{t}-D_{i}(t,T)}{C}+S_{t}\right)$$

where *D*_𝒾_(*t*, *T*) is the dose calculated using Equation 2, and *D_t_* ∈ ℝ*^N^* represents the target dose for each voxel, which is defined as


(6) $$D_{i}^{t}=\{\begin{array}{c} D_{o}^{t},i\in V_{o}\\ D_{t}^{t},i\in V_{t}\\ 0,otherwise. \end{array}$$

Here *D_o_* is the maximum dose that could be delivered to the *o-*th OAR while *D_t_* is the minimum dose that should be delivered to the tumor. Additionally, in Equation 4 and 5, *C* ⊂ ℝ is a constant that scales down the cost while *S_o_* and *S_t_* are constants that control the relative importance for the *o*-th OAR or the tumor, respectively. Unlike the general multi-objective optimization approach, in which a set of weighting parameters are utilized in presenting the total cost function as a convex combination of all the individual terms, horizontal shifting constants *S_o_* and *S_t_* are used to balance the relative importance of the individual cost functions. These cost functions are illustrated in **Figure 2B** which depicts cost functions for OARs (green) and the tumor (blue) depending on the calculated dose *D*_𝒾_. As shown in **Figure 2B**, a larger *S_o_* and *S_t_* implies more weight on the corresponding term, whether it is for an OAR or for the tumor.

The cost function presented in Equation 3 above may be expressed in a denser form to provide the optimization model given by Equation 7 below:


(7) $$\underset{t\in X,T\in Y}{\min}F(t,T):=\sum_{i\in V}f_{i}(t,T),$$

where


(8) $$f_{i}(t,T)=\{\begin{array}{c} f_{i}^{o}(t,T),i\in V_{o}\\ f_{i}^{t}(t,T),i\in V_{t}\\ 0,otherwise. \end{array}$$

Equation 7 includes two blocks of variables and is convex for dwell time t when fixing transmission rates *T*, but not for the alternate case for transmission rates *T* when fixing dwell time *t*. To show this, it can be verified that $f_{i}^{o}(t,T)$ is convex for one block of variables when fixing the other and $f_{i}^{t}(t,T)$ is convex for *t* but concave for *T*. Specifically, *D*_𝒾_ is a linear function of *t* so it is both convex and concave, but it is convex for *T* as the Hessian is positive semidefinite. On the other hand, $f_{i}^{o}$ is a convex non-decreasing function for *t* and *T* while $f_{i}^{t}$ is convex and non-increasing. Since the summation of convex functions is convex, it follows that *F* is convex for *t* but the convexity with respect to *T* is not clear.

In order to solve Equation 7 an alternating minimization scheme is utilized to search for t and T in turns. This alternating minimization algorithm starts at an initial point (*t*_0_, *T*_0_) ∈ 𝒳 × 𝒴 and solves the two sub-problems with respect to t and T while fixing the other by gradient descent with back-tracking line search. Specifically, the partial gradients of *f*_𝒾_(*t*, *T*) with respect to the *j* ^th^ dwell time is expressed as Equation 9 as follows:


(9) $$\frac{\delta }{\delta t_{j}}f_{i}(t,T)=\{\begin{array}{c} \frac{f_{i}(t,T)D_{l}^{J}T_{g(i,j)}^{\dot{\alpha }(i,j)}}{C},i\in V_{o}\\ -\frac{f_{i}(t,T)D_{l}^{J}T_{g(i,j)}^{\dot{\alpha }(i,j)}}{C},i\in V_{t}\\ 0,otherwise, \end{array}$$

and partial gradients of *f*_𝒾_(*t*, *T*) for the *k* ^th^ transmission rate *T*_k_ is expressed as Equation 10 below:


(10) $$\frac{\delta }{\delta T_{k}}f_{i}(t,T)=\{\begin{array}{c} \frac{f_{i}(t,T)D_{l}^{\dot{J}}t_{j}}{C},i\in V_{o},g(i,j)=k\\ -\frac{f_{i}(t,T)D_{l}^{\dot{J}}t_{j}}{C},i\in V_{t},g(i,j)=k\\ 0,otherwise. \end{array}$$

**Figure 3:**
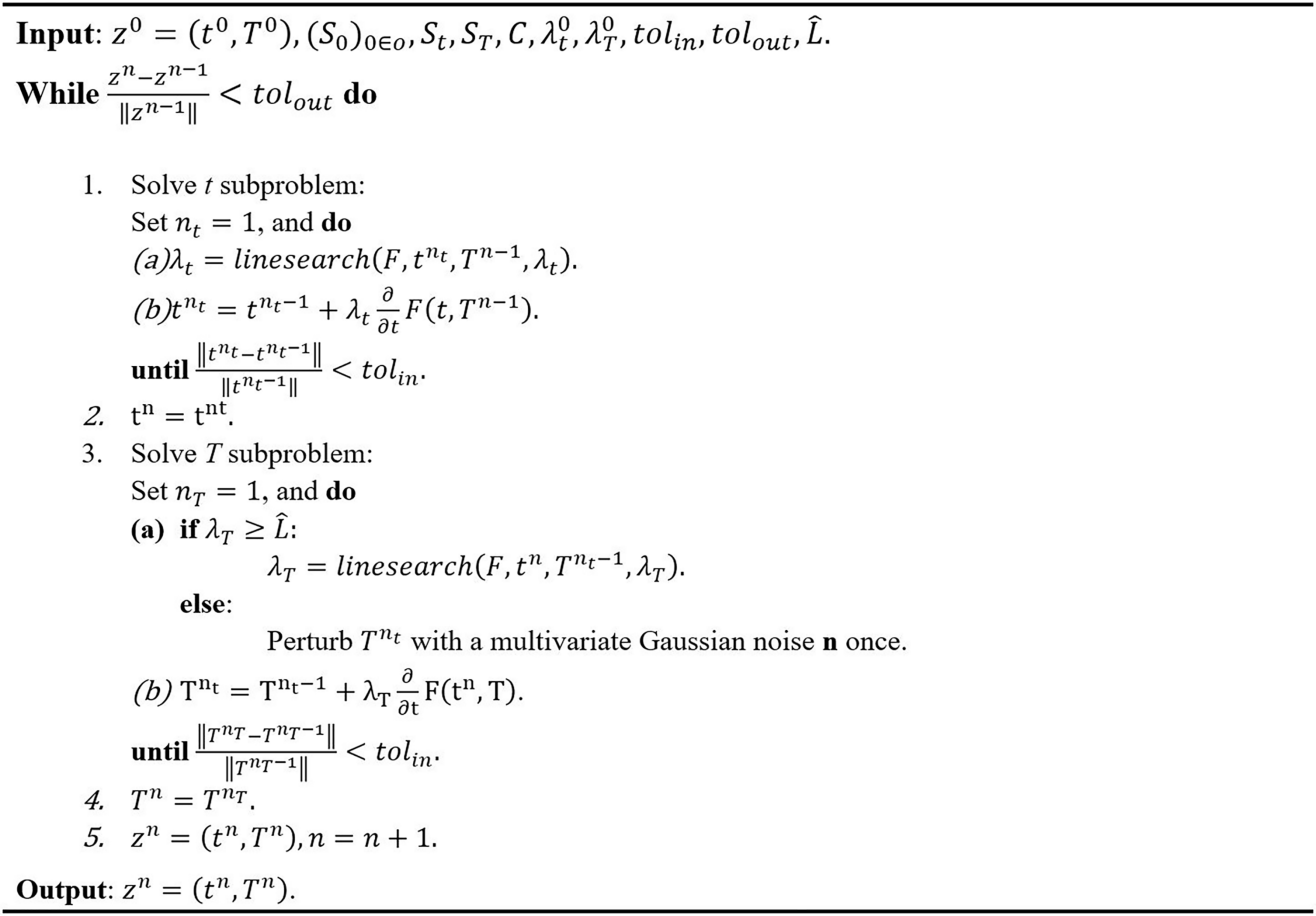
Alternating minimization algorithm for Equation 7.

The gradient *F(t, T)* with respect to *t* and *T* can be obtained by taking summations of Equations 9 and 10. The gradient descent method with constant step size bounded by $\frac{1}{L}$ is unlikely to converge to a strict saddle point, where *L* is the Lipschitz constant for a function *f* ∈ *C* ^2^. A random perturbation to the iterate *T_i_* may be made when the step size is sufficiently small, or approximately less than the Lipschitz constant, and then the constant step size may be used to continue the optimization. The scheme of the algorithm is summarized as shown in **Figure 3**.

**Figure 4:**
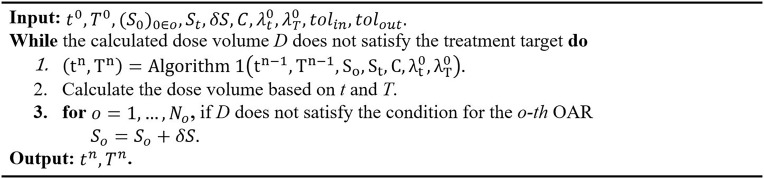
Automatic search algorithm.

For a user-specified set of constants *S_o_* and *S_t_*, the algorithm in **Figure 3** will generate a plan with dwell time *t* and transmission rates *T*. However, the estimated dose-volume histogram (DVH) associated with this generated plan may not satisfy the clinical goal for tumor coverage and OAR dose. For a reason, an automatic algorithm to tune the control constants *S_o_* and *S_t_* are introduced to generate a satisfactory plan. The algorithm assumes a relatively large *S_t_* initially to ensure that the OAR sparing fails for at least some OARs, indicating that the current weighting of the cost function favors tumor coverage. Subsequently, the algorithm gradually increases *S_o_* if the *o*-th OAR received an excessive dose, and performs an additional iteration with the updated *S_o_*. This procedure terminates when the OAR doses satisfy the prescribed criteria. This process of tuning the control constants *S_o_* and *S_t_* is summarized in **Figure 4**. An initial guess of *S_t_* used to tune *S_o_* and *S_t_* automatically in a manner similar to the algorithm.

### Validation of the Model

We first calculated the dose rate for the region of interest, followed by the dose rate calculation at the dwell position *p*, denoted by ${\dot{D}}_{p}$ . The dose can then be calculated by


(11) $$D_{p}(i)=\sum_{p=1}^{K}D_{\dot{p}}(l)\times t_{p}\times T_{g(i,p)}^{\alpha (i,g(i,p))}$$

**Figure 5:**
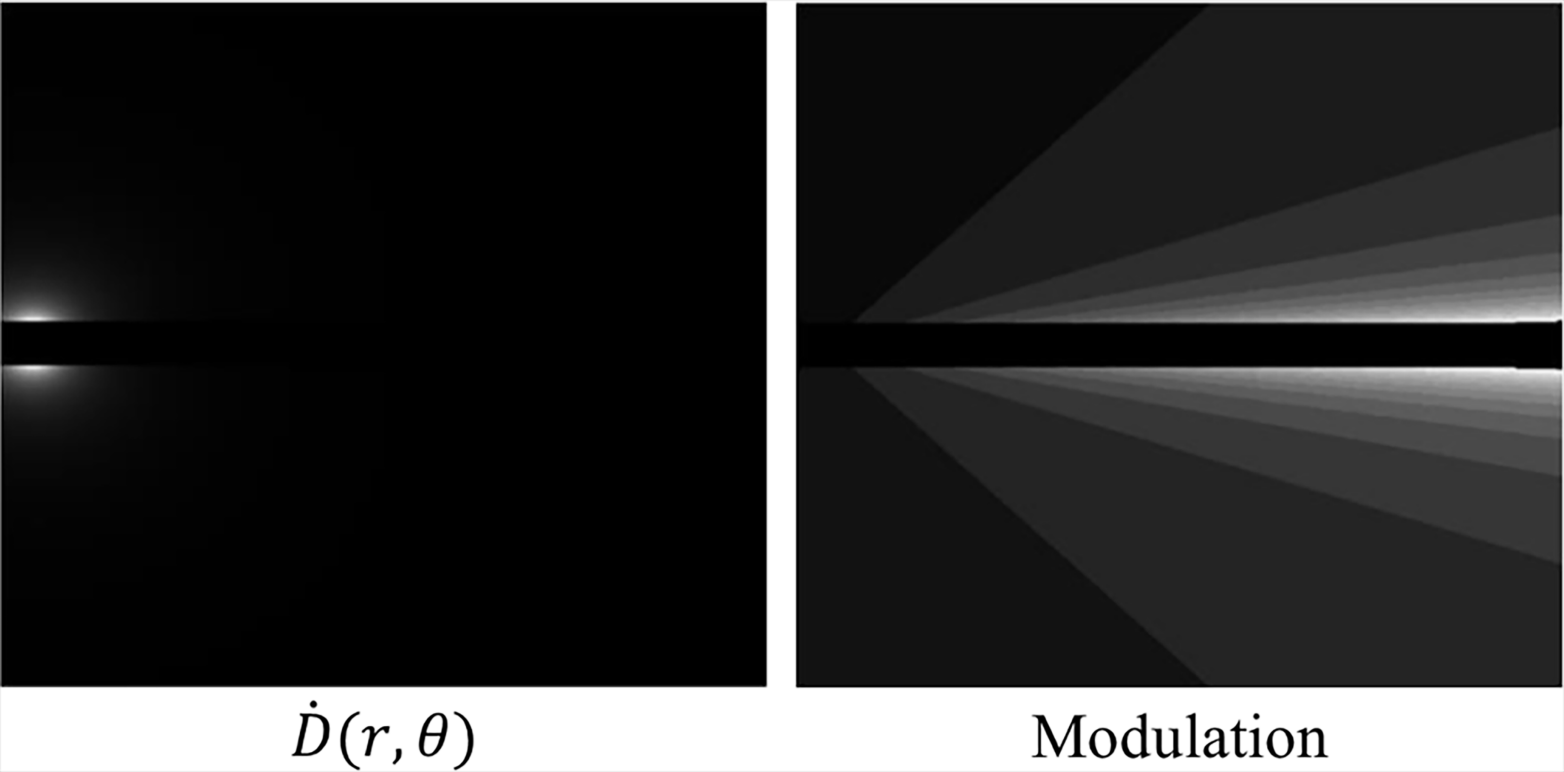
Left: A dose rate map at a first dwell position within our proposed method. Right: The modulation at a first dwell position within our proposed method. The lighter shades of grey indicate less modulation due to the anisotropic nature of the source.

Here *t_p_* is the dwell time at location *p* and *T* is the transmission rate for a shield indexed by a function *g*(*i*, *p*) that depends on the dwell position and the pixel location. *α*(*i*, *g*(*i*, *p*)) is a constant depending on the location of the pixel and the index of the transmission rate. The dose rate map and modulation at a first dwell position is shown in **Figure 5**. The cost function we are considering is given by


(12) $$f_{total}(X)=f_{organ}(X)+f_{tumor}(X)=\sum_{r=1}^{3}\sum_{i=1}^{N}e^{\frac{X_{r}(i)}{c}+Sr}+\sum_{i}^{N}e^{-\frac{X_{4}(i)}{c}+S_{4}}$$

where,


(13) $$X_{r}(i)=\sum_{p}^{K}t_{p}D_{p}(i)T_{g(i,p)}^{\alpha (i,g(i,p))}-D_{o,r}(i)$$

Finally, the partial derivatives with respect to the dwell time *t_p_* and transmission rate *T_j_* are given by


(14) $$\frac{\partial f_{organ}}{\partial t_{p}}=\sum_{p}^{K}\sum_{i}^{N}\frac{D_{\dot{p}}(l)T_{g(i,p)}^{\alpha (i,g(i,p))}e^{\frac{\Sigma_{p}^{K}t_{pD_{p}}(i)T_{g(i,p)}^{\alpha (i,g(i,p))}-D_{o,r}(i)}{c}+S_{r}}}{c}$$

and,


(15) $$\frac{\partial f_{organ}}{\partial T_{j}}=\sum_{p}^{K}\sum_{i}^{N}I(p,i,j)\frac{\alpha (i,g(i,p))D_{\dot{p}}(l)t_{p}T_{g(i,p)}^{\alpha (i,g(i,p))-1}e^{\frac{\Sigma_{p}^{K}t_{pD_{p}}(i)T_{g(i,p)}^{\alpha (i,g(i,p))}-D_{o,r}(i)}{c}+S_{r}}}{c}$$

where *I*(*p*, *i*, *j*) is an indicator function determining whether the *i*-th pixel will be affected by the *j*-th transmission rate at dwell location *p*.

We then alternately minimized the cost function with respect to one variable, fixing the other:


(16) $$t^{k}=\underset{S_{t}}{\mathrm{argmin}}f(t,T^{k}),T^{k}=\underset{S_{T}}{\mathrm{argmin}}f(t^{k+1},T)$$

where *S_t_* = {*t*|*t*≽0} and *S_T_* = {*T*|0.25≼*T*≼0.8}. For each sub-problem, we use the gradient descent with backtracking line search method. Upon convergence, the constraints for OARs are checked; if the constraints are not met, then the cost function is automatically modified by changing *s_r_* such that the OAR is favored.

### Evaluation With 2D Phantom and 2D Patient Cases

**Figure 6:**
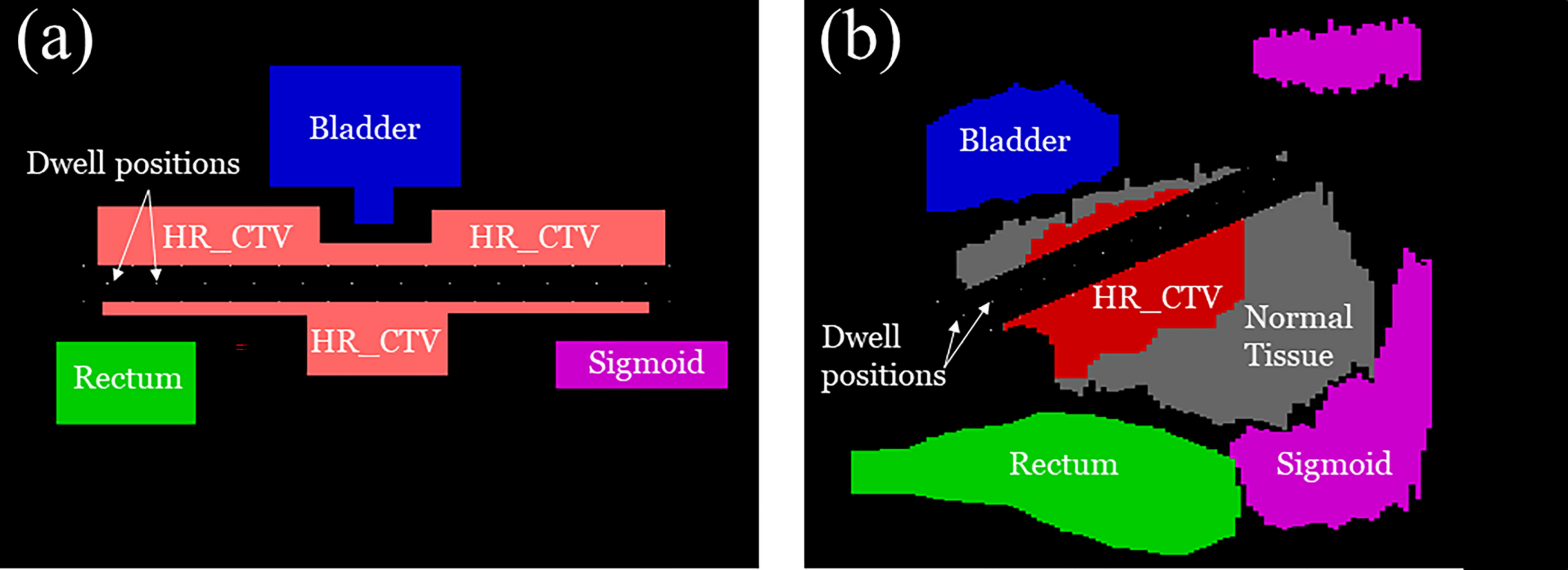
A schematic illustration of **(A)** 2D phantom and **(B)** 2D patient models used for the experiment including the clinical tumor volume (CTV) in relation to organ positions of the OARs.

We performed numerical experiments on the 2D phantom and 2D patient data which consisted of a HR_CTV positioned on both sides of the tandem applicator and three OARs; a bladder, rectum, and sigmoid. The details of each configuration are shown in **Figure 6**. With the patient data, which the dimension was 332×502×118 (resolution of 0.29 cm×0.29 cm×0.8 cm), when we implemented our method to calculate by setting CUDA C++ for parallel computation and optimize the treatment plan it only took around 1 minute. We employed CPU of Intel i7-6700k 4.00 GHz, GPU of Nvidia GTX 1080, and 32 GB DDR4 3200 MHz memory. To compare the proposed method with the conventional one, we use the same model and solver, but we fixed the transmission rates as constant (= 1.0). A radioactive Ir-192 source was utilized, and data was collected for the twelve dwell positions of the Ir-192 source that were monitored. The transmission rates and dwell times were calculated for each of the twelve dwell positions and compared between the conventional and proposed method. Similarly, the dose distribution and coverage statistics were also calculated for both methods.

## Results

### Non-Isotropic Dose Distribution

**Figure 7:**
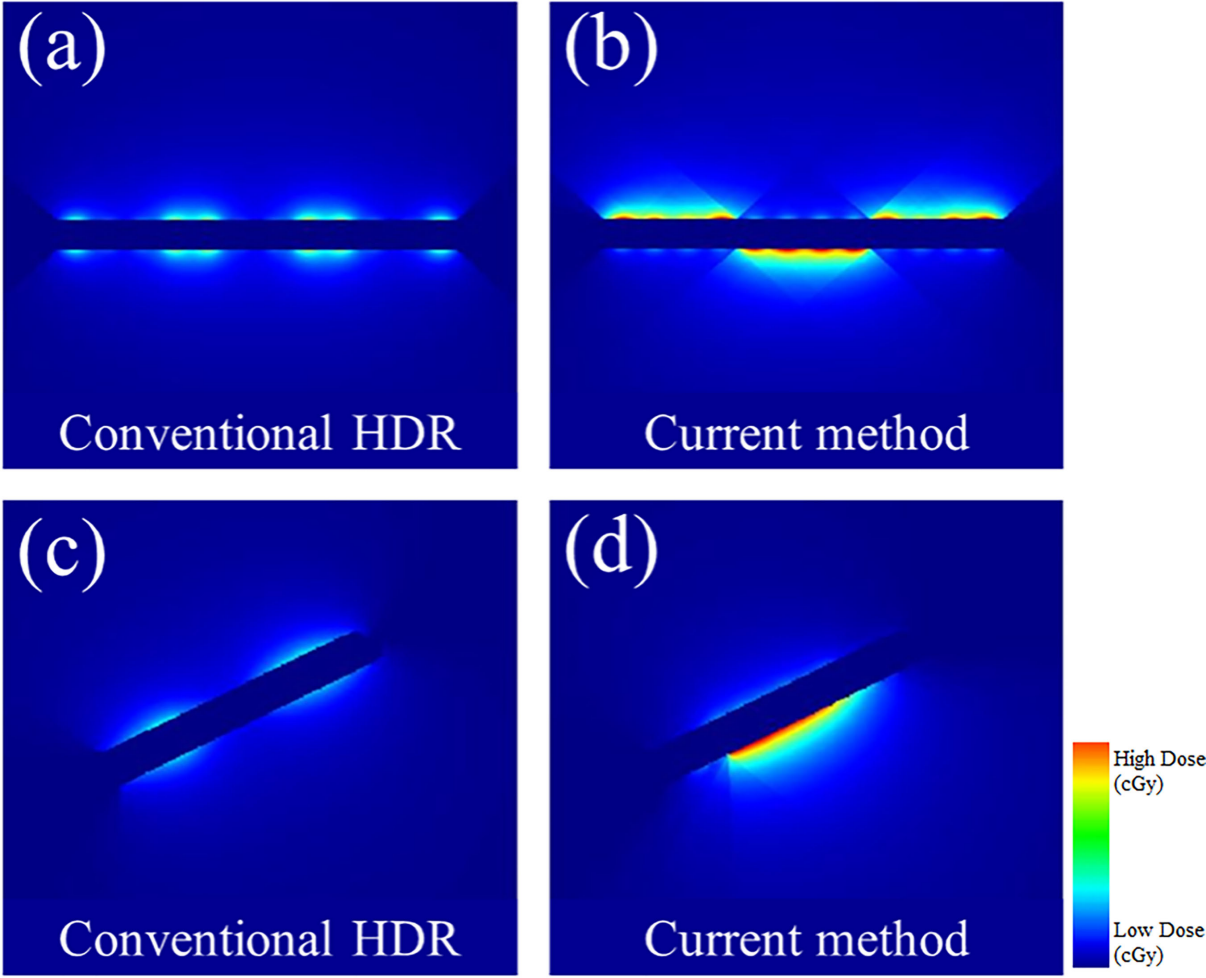
A comparison between the conventional HDR method and our proposed (current method) with the 3D metal printing for anisotropic field. **(A, B)**: The dose distribution estimated using the 2D phantom data. **(C, D)**: The dose distribution estimated using the 2D patient data.

**Figure 8:**
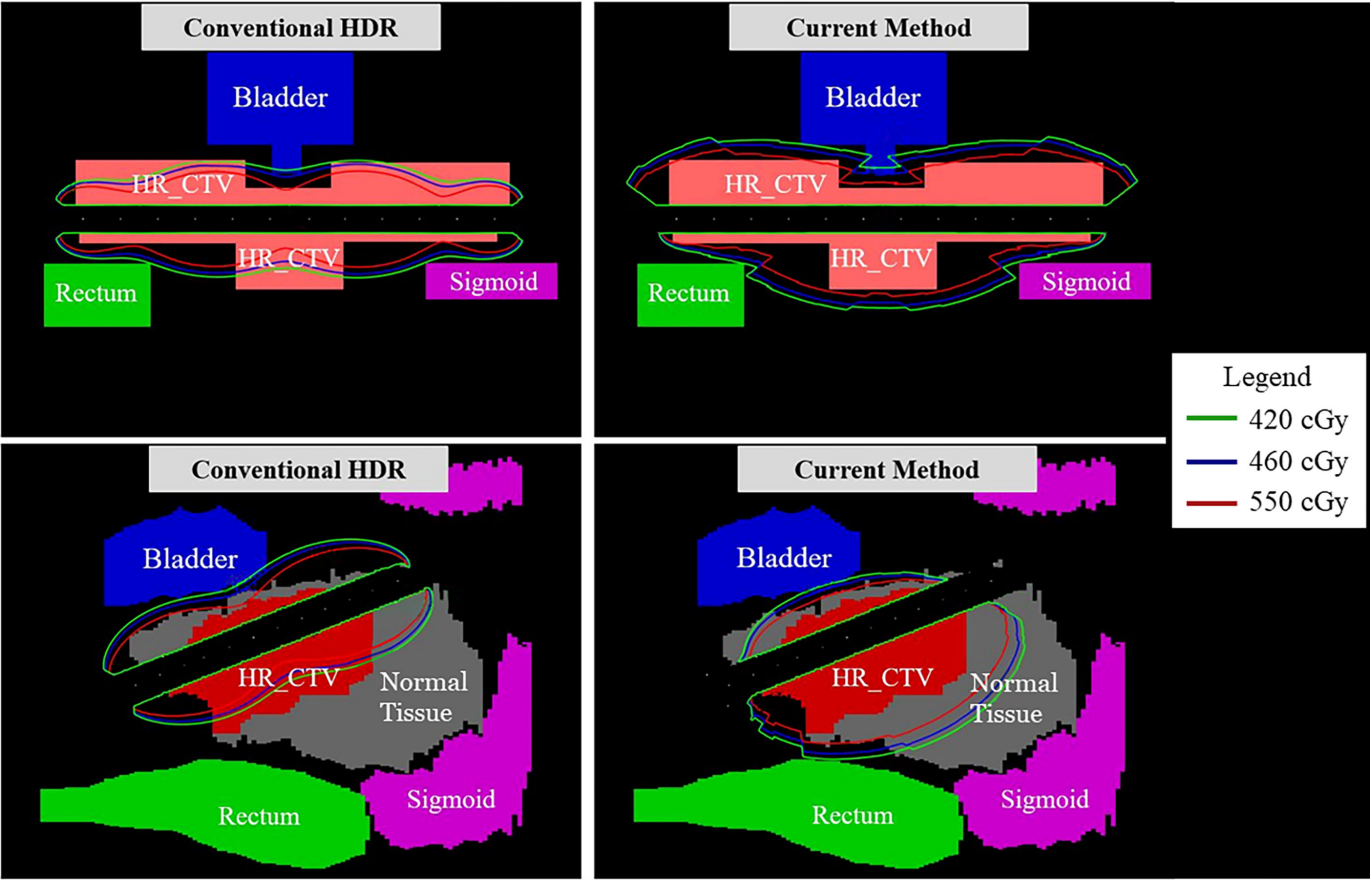
A comparison of the dose distributions between the conventional method and proposed.

In **Figure 7**, the observable differences in the intensity profiles of the current HDR method and our proposed method demonstrate the directional treatment capabilities of the patient-specific tandem applicator. The major advantage of our method possesses is the dose can be modulated at each dwell position based on the anatomical and geometrical information shown in both the 2D phantom and patient cases. The proposed method significantly improved the coverage by approximately 70% for the simplistic phantom case shown. **Figure 8** illustrates the dose distribution for each case by displaying the three important isodose lines: 420 cGy, 460 cGy, and 550 cGy, which correspond to the maximum dose for the rectum and sigmoid, bladder and the prescription to the HR_CTV, respectively.

### Transmission Rate and Dwell Time Comparison

**Figure 9:**
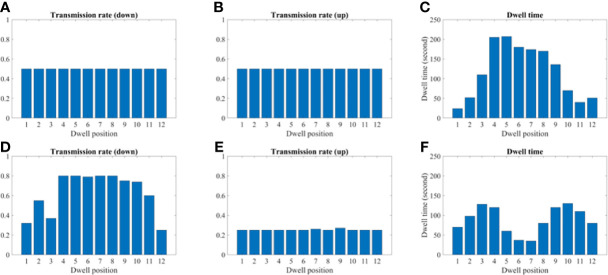
A comparison between the conventional method and proposed using the 2D phantom model. **(A–C)** A transmission and dwell time for the conventional method. **(D–F)** A transmission and dwell time for the proposed method. To compare the proposed method with the conventional one, we use the same model and solver, but we fix the transmission rates as constant (= 1.0).

**Figure 10:**
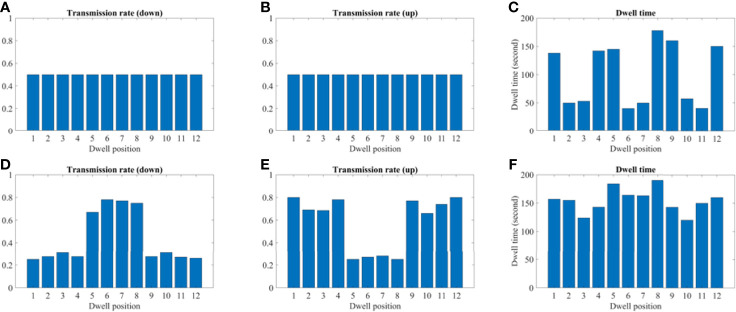
A comparison between the conventional method and proposed using the 2D patient model. **(A–C)** A transmission and dwell time for the conventional method. **(D–F)** A transmission and dwell time for the proposed method. To compare the proposed method with the conventional one, we use the same model and solver, but we fix the transmission rates as constant (= 1.0).

**Figures 9** and **10** show the comparison of transmission rate and dwell time between the conventional method and our proposed method for both the 2D phantom and patient cases. The dwell time for the patient case using the proposed method was 23.78 minutes at 6.4 Ci Ir-192 source strength. The proposed approach exhibited substantial improvements in HR_CTV coverage over the conventional method. A summary of changes to dose metrics when implementing the proposed device compared with the conventional applicator is provided in **Figure 3**. For the phantom case, 99.18% of the HR_CTV was covered by the prescribed dose using the proposed method, compared to only 58.32% coverage achieved by the conventional method. For the patient case, the proposed method increased the coverage of the HR_CTV from 56.21% to 99.92%. In each case, both methods satisfied the treatment constraints for neighboring OARs.

## Discussion

This study indicates a clear dosimetric advantage when comparing the proposed applicator with a traditional HDR tandem applicator for simulation studies. The validation of the algorithm for construction and dose calculation for the 2D cases is demonstrated with additional verification on clinical datasets required. The most impact of the designed applicator is additional sparing of OARs, while maintaining coverage of the HR-CTV. It has been found that in order to safely treat the patient by preventing high radiation exposure to surrounding tissues, including the bladder, rectum, and sigmoid, are often a limiting factor. 90% is typical minimum constraints for the HDR brachytherapy but there are some cases when rectum/bladder (especially rectum) being too close to the HR-CTV and the minimum coverage violates 450/4480 cGy D**_2cc_**. We had seen this anecdotally through our clinic’s cases. Nonetheless, our most cases are still clinically acceptable but can be improved were it not for OAR involvement.

Various attempts at modulating dose for different disease sites have been proposed and implemented since the introduction of 3D-guided brachytherapy. One such technique has a movable tungsten shield called paddle-based rotating shield brachytherapy (P-RSBT) (15). The method has an advanced feature in terms of shielding the source, but this takes more time than the conventional technique. A major concern with any procedure requiring anesthesia is the prolongation of treatment time. While longer than conventional treatments, our technique does not add a significant amount of time and is in line with other proposed IMBT methods. Our methodology while it can be utilized with model-based dose-calculation algorithms, also benefits from being compatible with TG-43 formalism, thereby reducing calculation time. Our design is similar to the static modulation designs that have been previously introduced. Static modulation has been much more common historically, ranging from shielded sources like CivaSheet to shielded applicators such as the shielded ovoid. However, these are more simplistic in their design and do not use optimization to tailor the dose to surrounding tissues further. The major advantage of our proposed applicator design is the increased dose modulation ability compared with static, fixed-geometry applicators, and even dynamic methods. The ability to use any angular position enables the dose to be uniquely conformed with the 3D printed applicator to each patient’s geometry.

**Figure 11:**
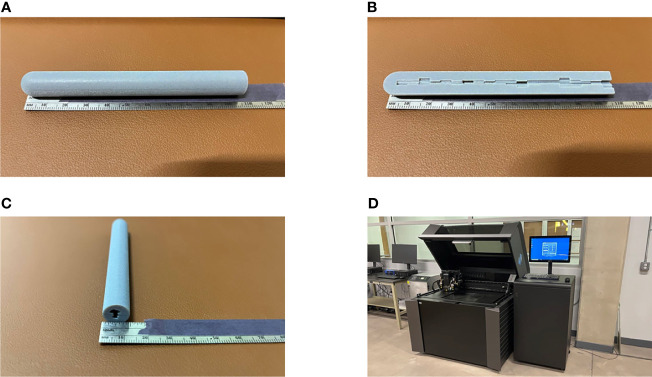
A prototype of our proposed IMBT printed by a 3D printer. **(A–C)** The actual printing that has a diameter of 1.2 cm and 10 cm long for illustrative purpose. **(A, C)** The whole piece of the IMBT tandem printed. **(B)** The half-cut of the printed IMBT tandem to visualize the inner shape. **(D)** The PolyJet print that we used for this study due to the fine resolution (30 µm) and the color availability (Pantheon 444C). It takes two hours to print and cured.

For our design, the maximum diameter of the applicator was limited to 1.2 cm in order to be feasible for insertion after cervical dilation, while also allowing enough thickness for tungsten shielding. The tandem diameter of 1.2 cm is large compared to current tandems and the goal was to create adequate attenuation, but this was based on previously used commercial LDR tandems that range from 6-8 mm in diameter. In future work we will continue to optimize to determine a clinically significant dose reduction while minimizing the tandem size. However, we do expect that the tandem will be larger than current HDR tandems and will require anesthesia and dilation. For the simulation, only the length of the tandem that would be used for shielding was considered in the design, however the whole length of the tandem can be constructed using the same 3D metal printing technique with a fixed inner diameter. Unlike a general 3D printer, which either uses a fused deposition modeling (FDM) or stereolithography (SLA) type, the 3D metal printer requires a sintering process after printing (16, 17). During the process, the surface finish and tolerances may not be the same as those of machined components; typically, the 3D metal printer can achieve around 10.0–12.0 µm resolution. The 3D printed tungsten is granular, so the surface is less smooth than a machined surface. Because of this granularity and also because of the inherent difficulty in printing overhangs, it is still a challenge to ensure how accurate the detail of the internal bore would be. Hence, quality assurance procedures will need to be used to test the positional accuracy on a daily basis. In addition, each section of the tandem would need to be inspected and compared to the calculated thickness to ensure accurate printing and alignment. Dosimetric verification of the proposed remains the subject of future work. Despite the difficulties of the 3D metal printing, once the user can determine the optimum build operation, it could be used in a clinic similar to other clinical 3D printed plastic applicators. Additional work must be completed to construct a prototype version of the tandem based on 3D patient anatomy for extensive dosimetric measurements. The 3D printed design demonstrated here is for illustrative purposes and was manufactured with a resilient polymer called Vero material with PolyJet printing (J850, Stratasys, Eden Prairie, MN). For comparison, PolyJet type has a 32 µm resolution in all x, y, and z directions, so the accuracy of the 3D metal printed design will be an order of magnitude more precise. **Figure 11** shows our prototype and printer that we used for this study. It is the goal to further utilize our design and print it out as a full-length tandem for further phantom studies. However, a dosimetric analysis of that prototype is beyond the scope of this paper.

This study has explored the feasibility of use of 3D metal printing for HDR IMBT. Recently, 3D printing technologies have been increasingly adopted in various medical physics fields including electron and photon beam therapy, immobilization device, and brachytherapy. Current research has been limited to making 3D printed models using PLA or other similar plastics. There is increasing interest in incorporating metallic materials in 3D prints, however that has been limited to using either metallic powders or ball bearings (18, 19). Going forward, we will verify the dosimetric properties and the reproducibility of the manufacturing process. To be used clinically, a highly precise mechanism will need to be in place to ensure the positional accuracy of the delivery and insertion of the tandem. As with other applicators, device orientation will need to be confirmed before each treatment.

## Conclusion

The present study demonstrates the simulation of an inverse plan-based IMBT using a tungsten printed geometry using a modified TG-43 methodology. It is anticipated that our method would be a good alternative to conventional applicators when surrounding OARs limit the tumor-dose coverage. Moreover, this method could be adapted to other HDR sites such as rectal, vaginal, prostate, and breast.

## Data Availability Statement

The original contributions presented in the study are included in the article/supplementary material. Further inquiries can be directed to the corresponding author.

## Author Contributions

JS mainly simulated and wrote the manuscript. MP did English proofread and corrected the flow of the manuscript. S-WK analyzed some of the results. W-HK and Y-HC modified the equations and put an idea in the discussion part. J-BC and K-YE managed all related studies and supported them. All authors contributed to the article and approved the submitted version.

## Funding

This research was supported by the Mid-career Researcher Program (Grant No. 2018R1A2B2005343) through the National Research Foundation of Korea and by the Seoul National University Bundang Hospital (SNUBH) Research Fund (Grant No. 14-2021-014).

## Conflict of Interest

The authors declare that the research was conducted in the absence of any commercial or financial relationships that could be construed as a potential conflict of interest.

## Publisher’s Note

All claims expressed in this article are solely those of the authors and do not necessarily represent those of their affiliated organizations, or those of the publisher, the editors and the reviewers. Any product that may be evaluated in this article, or claim that may be made by its manufacturer, is not guaranteed or endorsed by the publisher.
